# Sexual health and wellbeing training with women in Pacific Island Countries and Territories: a scoping review

**DOI:** 10.1080/16549716.2021.1948673

**Published:** 2021-07-29

**Authors:** Nalisa Neuendorf, Karen Cheer, Rachael Tommbe, Clare Kokinai, Lalen Simeon, Kelwyn Browne, David MacLaren, Michelle Redman-MacLaren

**Affiliations:** a College of Medicine and Dentistry, James Cook University, Cairns, Australia; b Papua New Guinea Institute of Medical Research, Goroka, Papua New Guinea; c The Cairns Institute, James Cook University, Cairns, Australia; d School of Health Science, Pacific Adventist University, Port Moresby, Papua New Guinea; e School of Arts and Humanities, Pacific Adventist University, Port Moresby, Papua New Guinea; f Deputy Vice Chancellor, Chancellory, Pacific Adventist University, Port Moresby, Papua New Guinea; g Independent Scholar, Port Moresby, Papua New Guinea

**Keywords:** Sexual health, training, women leaders, pacific islands, papua new guinea

## Abstract

**Background:**

Women who are spouses of students at a faith-based university in Papua New Guinea (PNG) are afforded proximal power. These women are perceived as leaders and regularly approached by members in their communities to provide advice on sexual and reproductive health matters. Women leaders therefore need access to sexual health information and training to provide appropriate advice.

**Objective:**

The aim of this paper is to review the characteristics of community-based sexual health training in Pacific Island Countries and Territories (PICTs), as reported in published literature. This is evidence to inform the development of sexual health training programs for women in PNG.

**Methods:**

A systematic search of databases, repositories and websites identified peer-reviewed studies. Grey literature was also sourced from government and non-government organisations and PNG health professionals. Six published papers, one report, one health worker practice manual and one health worker training package were identified for inclusion. Selected papers were assessed against the Canadian Hierarchy of Evidence to determine quality of evidence for practice. Themes were identified using a thematic analysis approach.

**Results:**

Three themes became apparent from the literature synthesis: i) *program development*; ii) *mode of delivery*, and iii) *evaluation*. Social and cultural context influenced all elements of sexual health training in PICTs. Few studies reported evidence of comprehensive evaluation.

**Conclusions:**

Successful sexual health training programs in PICT communities are designed and delivered accounting for local contexts. Programs that engage participants with diverse abilities inspire change to achieve desired outcomes. Key findings from this study can be used to assist women leaders to contextualise and operationalise sexual health training to promote the wellbeing of members in their communities.

## Introduction

The Pacific Region comprises 22 diverse countries and territories, covering Melanesia, Micronesia and Polynesia [1]. Women in these Pacific Island Countries and Territories (PICTs) are overwhelmingly resourceful, highly skilled and have strong social bonds in their home environments [2]. Wellbeing within this setting is based around the notion of relational harmony – symmetry in relationships between an individual, their family and community, and the natural and spiritual world [3,4]. The private spaces of the home environment are framed within and reinforced by these important relations that often afford women great agency. Although women may have a strong influence in the private space, this influence is not always extended into the public space [5]. In some PICTs, this is attributed to particular cultural norms [6]; in other PICTs, the colonising influence of Christianity has diminished the previously valued customary role of women in the public domain [7,8]. In Samoa for example, the public role of the *feagaiga*/sister has been downplayed, while maternal and domestic roles have been encouraged in the private sphere [9].

The individual and collective power of women in PICTs is increased by their access to formal and informal education, yet educational opportunities are often limited for girls and women [10,11]. Education for women is not without risk, as the presence of educated women can disrupt the predominantly patriarchal social order and associated structures [12]. There is an urgent imperative to consider how women can enhance and extend their positions of influence from private to public spaces in meaningful, appropriate, realistic and safe ways, through the acquisition of knowledge [13].

Education is highly valued by most Pacific peoples. In one Pacific nation, Papua New Guinea (PNG), proximity to education can afford proximal power [13]. In a recent study conducted in PNG, women who are spouses of students at Pacific Adventist University (PAU) stated they are regularly sought out to provide advice on matters of sexual health and wellbeing by other women in their home environments and in the institutional space [2]. We define sexual health as encompassing not only reproductive health but also sexual relationships, sexual assault and violence, mental health, gender identities and sexual orientation [14]. As spouses of graduate health workers, teachers and pastors, these women are considered knowledgeable advisers in the communities they either return or to which they become responsible. Their proximal power exists as an extension of their husband’s responsibilities. They are perceived as powerful women because they are married to an educated person or have lived in a *ples bilong save* (English: a place of knowledge/learning) i.e. a university campus. In the cultural context of PNG, which includes such concepts as *wantokism (*English: related through social or cultural affiliations); *pasin* (English: behaviour) and *luksave* (English: perception) [15], power and status are associated with and afforded due to the acquisition of knowledge through education. People with status and power hold great responsibility and are expected to provide advice when requested [16,17].

## Rationale for the review

Women spouses at PAU preparing to return to remote village communities are trusted as *meri lidas* (English: women leaders), as it is assumed they are the best point of access to information and knowledge surrounding sexual health and wellbeing issues [13]. Strong customary practices, social bonds and introduced Christian faith in rural and remote locations, along with limited access to health care services and quality information, offer little opportunity to learn about or respond to sexual health and wellbeing issues, particularly for women [18–20].

*Meri lidas* at PAU requested training and support for assuming a leadership role in the sphere of sexual health and wellbeing. Specifically, *meri lidas* requested further knowledge and focused skills about sexually transmitted infections (STIs) including HIV and AIDS, sexual practices, sexual hygiene and sexual decision-making/autonomy [2]. The women explicitly stated that their families and communities expected them to have knowledge of various sexual health issues. As such, they had a high desire to prepare for their postings to remote, resource-limited PNG villages [2].

## Aim and objectives

The review aimed to document and evaluate the nature and quality of community-based training reported in the literature that promotes the sexual health and wellbeing of communities in PICTS. The objective of the review was to identify the initiating and facilitating environments, strategies, and outcomes of identified interventions. The purpose of the review is to inform development of an evidence-based training program for implementation with women in the Pacific context.

## Methods

A scoping review is an approach to evidence synthesis that systematically examines key concepts in existing literature and identifies knowledge gaps [21]. Scoping reviews are also useful to determine the types and diversity of evidence on a topic to inform research and practice [22]. In this paper, a scoping review method was considered appropriate to map community-based sexual health training in PICTs: a complex area of research that, to the knowledge of the authors, had not been previously reviewed [23]. A scoping review protocol was developed to identify and assess a wide range of materials to assist in developing appropriate training programs in the Pacific. The protocol outlined the methods of the search, selection criteria, analysis and assessment of the literature.

## Search strategy

Databases including MEDLINE, Cumulative Index to Nursing and Allied Health Literature (CINAHL), Informit and Scopus were systematically searched using a combination of keywords and database-specific subject headings (see Appendix A for the MEDLINE search strategy). Database searching was supplemented by citation searching of retrieved papers. The search for grey literature [24] was conducted across 12 websites that were by known by authors to have a clear focus on health-related training in PICTS. Selected grey literature was downloaded from organisational websites. Authors also contacted government and non-government organisations, as well as experts in the field, for additional literature. Grey literature reference lists were scanned and relevant resources downloaded. This process was undertaken concurrently with database searches.

## Applying inclusion and exclusion criteria

Inclusion and exclusion criteria were applied to the retrieved material. Literature was included where:

- published after 2002. In 2002, the World Health Organization (WHO) convened an internationally significant meeting, ‘Challenges in sexual and reproductive health: Technical consultation on sexual health’. Conducted in collaboration with the World Association for Sexology, delegates explored sexual health and wellbeing in response to ‘dramatic changes in understanding of human sexuality and sexual behaviour’ [25,p.1].
- published in English, or a Melanesian *lingua franca* (Tok Pisin, Pijin, Bislama or Fijian languages)
- publications focused on sexual health, training and PICTs

Publications focusing on school educational programs were excluded. Accessibility of papers was reliant on availability via institutional database subscriptions.

## Screening

**Figure 1.**
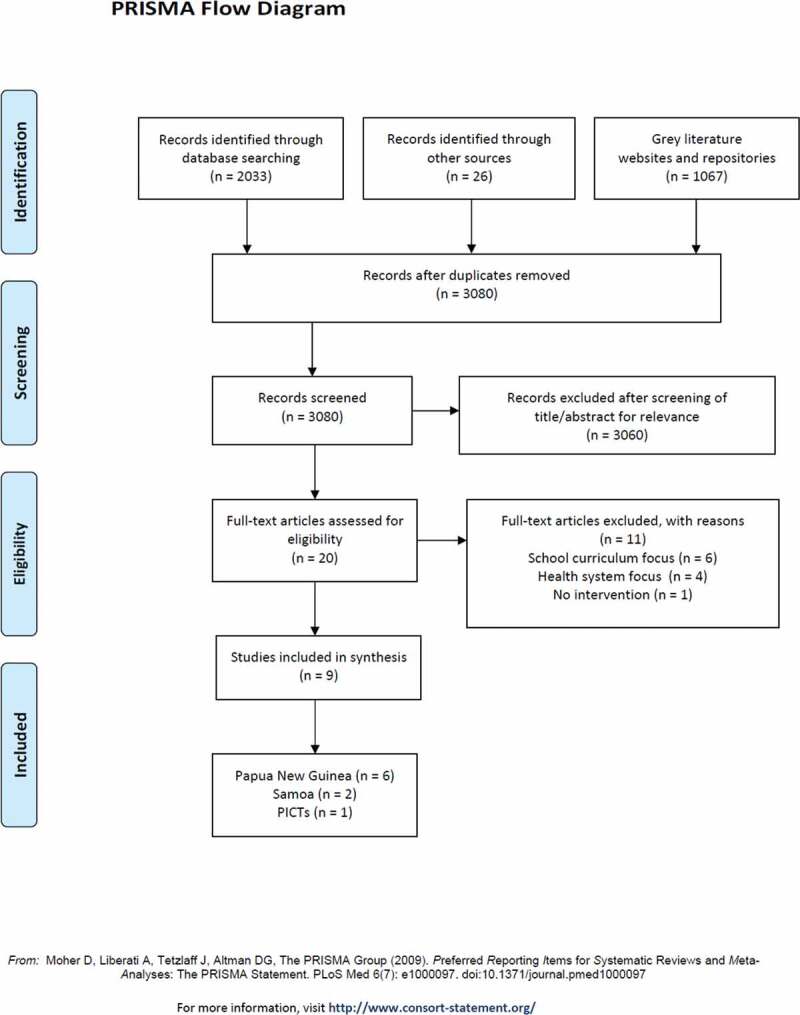
PRISMA summary [26]

The titles and abstracts of 3080 publications were screened by co-authors NN and KC, resulting in exclusion of 3060 publications. Rigorous full-text assessment of the remaining literature was conducted independently by two reviewers (MRM and KC) to ensure quality in the selection process. Authors conferred on literature selection until consensus was achieved. Nine publications were included. Figure 1 summarises the search strategy used for this review.

## Quality assessment

**Figure 2.**
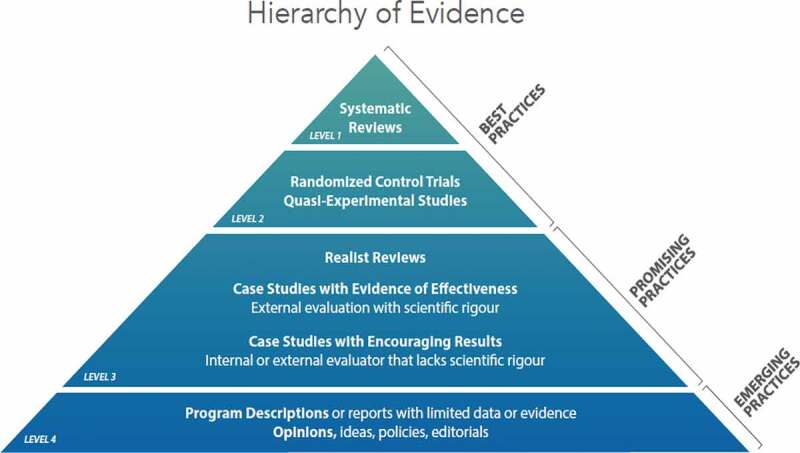
Hierarchy of evidence [27]

As the review focused on the characteristics of sexual health training interventions, the authors assessed the literature against the Canadian Hierarchy of Promising Practices Evidence [27] to determine quality (Figure 2). This hierarchy situates relevant literature into three categories and four levels of evidence, from best practice to promising practices and emerging practices. Developed for use in the social housing sector to evaluate program interventions in regards to ‘what works, why it works and for whom it works’ [27,p.4], the framework has also been used to assess evidence of the effectiveness of health care interventions [28–30].

Levels 1 and 2 of the hierarchy represent best practice, where rigorous scientific research has proven the intervention to be effective. Level 3 represents an intervention regarded to be a promising practice, where there is sufficient evidence to claim the practice is effective in achieving a stated aim or outcome, consistent with the goals and objectives of the program or activity. Promising practices ideally demonstrate effectiveness through rigorous scientific research; however, insufficient generalisable evidence exists to label these as ‘best’ practice. These practices show promise for other organisations and entities that wish to adapt the approach based on the trustworthiness of the evidence. Level 4 represents emerging practices, interventions that are novel and hold promise based on evidence of effectiveness or change that is not research-based and/or adequate to be deemed a ‘best’ or ‘promising’ practice. This may be because the intervention is new and there has been insufficient time to generate compelling results. Yet information regarding such interventions is important as it highlights innovation and emerging practices worthy of more rigorous research.

## Results

Nine publications met the inclusion criteria of the scoping review: six peer-reviewed papers; one health worker practice manual and one village health worker training package. With limited published peer-reviewed literature directly associated with sexual health training programs in PICTs, grey literature sources were important points of reference to understand the scope of sexual health training in the Pacific. Table 1 presents a summary of the included literature. Application of the hierarchy of evidence identified five publications as Level 3 Promising Practices and four publications as Level 4 Emerging Practices (Table 2).

**Table 1.** Summary of included literature

Author, year	Setting	Population	Focus area	Program
Baldwin, 2010 [38]	Papua New Guinea	Community	HIV	Performance drama
Barcham et al., 2016 [37]	Papua New Guinea	Volunteer health workers	Health promotion	Training program
Haseman et al., 2014 [39]	Papua New Guinea	Community	HIV	Folk opera
Heard et al., 2015 [40]	Samoa	University students	Sexually transmitted infections	Health promotion event
Heard et al., 2019 [32]	Samoa	Young people	Intimate relationships	Interactive theatre
National Department of Health Papua New Guinea, 2003 [33]	Papua New Guinea	Village health volunteers	Health worker training	Teaching and learning aids
Natoli et al., 2011 [34]	Papua New Guinea	Community	Sexually transmitted infections (STIs)	Village-level health promotion
Population Services International, 2011 [35]	Papua New Guinea	Married couples	HIV and other sexually transmitted infections	Marital relationship training and program evaluation
Secretariat of the Pacific Community, 2015 [36]	PICTs	Health workers	Sexual and reproductive health	Training manual

**Table 2.** Hierarchy of evidence of promising research practices

Author, year	Hierarchy of Evidence	Rationale
Baldwin, 2010 [38]	Emerging practice	Innovative drama performance intervention to address the gap between awareness and behaviour change regarding sexual health/HIV requires documented evidence of impact
Barcham et al., 2016 [37]	Promising practice	Empowerment-based training program in a resource-limited setting was a local initiative focused on strengths of the individual and community. Provides some evidence for intervention (program) effectiveness although high level evidence required
Haseman et al., 2014 [39]	Emerging practice	Locally-developed folk opera incorporates narrative drama performance with culturally specific poetry, song and dance. May be adapted for health promotion and social justice in other performative cultures. Requires evidence of impact
Heard et al., 2015 [40]	Promising practice	A health promotion initiative used existing social structure inclusive of religious and cultural considerations to promote good relationship and sexual & reproductive health messaging. Comprehensive evaluation of impact required
Heard et al., 2019 [32]	Promising practice	Locally-developed interactive theatre intervention engaged young Samoans in dialogue to better understand social and cultural influences on intimate relationships. Issues of trust and gender were explored. Further evidence of drama as an educational tool for addressing IPV required
National Dept. of Health, 2003 [33]	Emerging practice	Locally-specific program trains volunteer health workers to engage with their community to deliver sexual and reproductive health education and basic medical care. PNG-wide program with candidates assessed against national competency standards is ongoing. Manual only, requires evidence of effectiveness
Natoli et al. 2011 [34]	Promising practice	Approach underpinned by community development principles. Combination of training and an understanding of complex local issues regarding STI transmission and treatment seeking behaviour enables deeper community engagement with health services and positive health outcomes. May be adapted for other PNG initiatives
Population Services International, 2011 [35]	Promising practice	Small-scale program aligned with traditional customs to bring about behaviour change in relation to gender based violence, sexual concurrency and HIV prevention. Participant-led change resulted in improved marital relationships, family wellbeing and reduced HIV transmission risks. Further evaluation needed to adapt and scale-up the project for delivery across PNG and the wider Pacific region
Secretariat of the Pacific Community, 2015 [36]	Emerging practice	Training manual tailored to the Pacific context incorporates a holistic, rights-based framework to address diverse sexual and reproductive health needs. Activities provide opportunity for participants to better understand concepts and develop practical strategies to advance sexual and reproductive health rights for all members of the community. Manual only, evidence of effectiveness required

**Figure 3.**
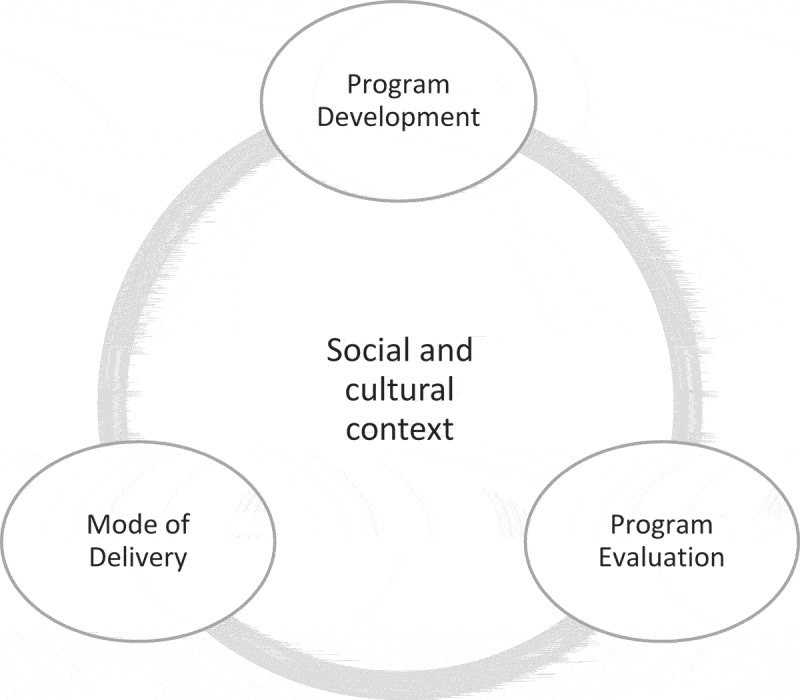
Centrality of social and cultural context to program themes

Drawing on the principles of inductive thematic analysis [31], results of the literature synthesis are framed within three key themes: i) program development; ii) mode of delivery, and iii) program evaluation. Each program theme is informed, enacted and connected through the social and cultural context (Figure 3).

## Program development

Informed by the review literature, we determine program development as the process of planning an effective program to address identified sexual health concerns. The need to consider social systems and cultural practices in training program development was evident across the reviewed studies. Gender and religion are two aspects that influence and mediate behaviours [32]. The local intrigues of society and culture can place individuals in vulnerable positions if not appropriately addressed.

### Gender

Cultural constructs of gender determine social roles and expectations for men and women in PICTs [32–34]. While some women have agency, in predominantly patriarchal cultural contexts, men are typically in public positions of power, privilege and control [32,33,35,36]. Gender inequality underpins poor sexual and reproductive health (SRH) for Pacific women [36]. Historical and contemporary cultural practices including bride price, early or forced marriage, and sexual violence are often mechanisms to disempower women and control their SRH [33,36]. Community members who identify as lesbian, gay, bisexual, transgender, queer and intersex (LGBTQI) also face challenges because of their gender identity, gender expression and sexual orientation: discrimination, stigma, violence, and lack of autonomy regarding SRH issues were reported as barriers that restrict LGBTQI persons’ access to sexual and reproductive health and rights (SRHR) [36]. Notions of gender can directly impact the mode of interaction and the transfer of knowledge within training programs [34,37]. Incorporating culturally specific, gender appropriate modes of interaction are important considerations when developing training programs that discuss sexual health topics and incorporate SRHR for diverse population groups in PICTs [32,34,36–40].

### Religion

There are diverse forms of religious expression among Pacific countries; yet free expression of sexuality and fulfillment of sexual and reproductive health is constrained by traditional patriarchal cultures within contemporary religious practices, commonly conservative Christianity, whose ideology and structure significantly shape daily life in PICTs [36]. The overarching frame of morality associated with religion and religious practices in PICTs may constrain access to sexual and reproductive health services and the content and reach of training programs [35,36]. This was reported as dependent on the beliefs of individuals in positions of authority within the church: church leaders in PICTs both supported [34,39], and opposed [40], sexual health programs. In their training, Village Health Volunteers (VHVs) were encouraged to work in partnership with church-based organisations to coordinate community health activities [33]. Religious institutions may permit sexual health training in church-owned facilities, particularly when no other venues are available [35]. In addition, all aspects of life for people in PICTs have a spiritual dimension; Christianity is often combined with traditional beliefs including sorcery [35–37]. The Christian Church thus presents as a space of both opportunity and challenge for program development in the sexual health domain. Training materials developed with respect of local social and cultural characteristics such as gender and religion were viewed as positively impacting the delivery of SRH training programs.

## Mode of delivery

The mode of implementation is important for sexual health programs. Identifying the best mode of delivery is contingent on the affect the program is likely to have in specific settings with a specific group, the type of training materials used, and the knowledge and skill of training facilitators [35]. Training programs should be flexible and responsive to the social and cultural considerations of local communities including the way people interact, and the language and terminology they use [37].

### Literacy

Literacy levels vary across the Pacific, and are essential to consider when delivering training programs [36,38,39]. Overcoming literacy barriers is necessary to engage facilitators when delivering training, as evident in the PNG participant training manual for VHVs, a primarily graphic representation of village life and health issues [33]. Similarly, in the *Life Drama* program in PNG, trainers who were illiterate and/or could not read English were encouraged to find other methods to remember training activities, including asking peers to translate and notetaking in *Tok Pisin*, a lingua franca of PNG [38]. Further, *Life Drama* as an educational strategy aimed for impact through delivery that did not rely on the written word to engage and inform audiences about sexual health [39].

### Language and meaning

In the *Stret Tokers* (English: straight talkers) program, to normalise conversation and promote behavioural change regarding sexually transmitted infections (STIs), the *stret tokers*/community engagement workers (CEWs) were encouraged to describe sexual and reproductive parts of the body in *stret* language, rather than use colloquial terms that may confuse the health message or reinforce harmful social norms that create barriers to accessing appropriate care [34]. Conversely, in the *Tokaut na Tokstret*! (English: talk out and talk straight) marital training program, participants were embarrassed and disturbed when descriptions of human anatomy and the reproductive system were not phrased as euphemisms or in parables [35]. The use of medical terms was viewed as challenging customs and may limit participant understanding of the discussion and the creation and retention of new knowledge [35]. Familiarity of trainers with community is culturally valuable as a precursor to sensitive discussions about sexual issues, and would ease anxiety when training begins [35]. Sexual health training programs in PICTs require facilitators who have a strong understanding of local contexts, who are culturally, socially and linguistically embedded in communities and have the necessary skill set to provide adequate training within these programs [33,38,39].

### Dramatic expression

Many sexual health awareness campaigns have been one-way dissemination of information, where the recipient does not engage with the message or adapt it to personal circumstances thereby reducing the probability of desired behavioural change [38]. Using appropriate forms of expression can engage people and enhance the reach of sexual health training programs. Several papers described drama-based interventions with participatory elements [32,38,39]. *Life Drama* is a workshop-based, participatory model of applied theatre and performance in PNG that emotionally engages participants in discussion and role play about the risks of HIV and provides them with accurate information in a meaningful way. The workshop group then situates an ‘open story’ about the consequences of HIV infection within the local context of family and community, for presentation in their communities [38]. To enhance engagement and make the learning experience more meaningful for performers and audiences alike, the *Life Drama* model was developed to incorporate additional Indigenous modes of performance, with songs, rituals and dances from regions across PNG [39]. Folk opera promoted the type of discussion and exchange of disparate knowledge that can propel social change [39]. Participatory theatre was used in Samoa with young people to highlight how multiple yet intersecting identities within social and cultural systems of power [e.g. gender, age, religion, sexuality] influence people’s sexual health and sexual behaviours [32]. Interactive engagement with the production *Suiga/Change*, provided participants the opportunity to voice varying interpretations and explore consequences and options for people situated in differing social positions [32]. In each of these studies, the mode of delivery provided space for individuals and groups to examine and question information, translate existing and new knowledge and embody new approaches to sexual health and wellbeing.

### Mixed modes

A combination of theoretical information and practical demonstrations were reported as beneficial to engaging participants and delivering successful training programs. The *Love Bugs* program in a Samoan university chose events, workshops and demonstrations to deliver sexual health information appropriate for the tertiary educational environment within which training was delivered [40]. The non-confrontational, mixed mode of delivery created a safe environment where young people were engaged and able to discuss sexual health, promote good relationships for positive sexual health outcomes, and share important sexual health information that is often stigmatised and considered taboo outside of this space [40]. In the *Stret Tokers* program, CEWs developed innovative ways to communicate sexual health information with their community, including story-telling, theatre and personal statements, combining these with practical demonstrations such as condom use [34]. Aware of existing gender inequity, the *Stret Tokers* worked in gender-balanced teams to model active participation of men and women [34]. Modes of delivery tailored to the unique contexts of PICTs, were participant-centred and culturally and socially appropriate, maximised participant engagement and were vital to program success.

## Evaluation

Program evaluation is necessary to determine the effectiveness and efficacy of the sexual health program. Given few reviewed publications provided rigorous evidence of evaluation, the level of evidence framework [27] was used to assess programs as emerging or promising practices for sexual health training in PICTs (Table 2). Programs were implemented in practical ways to achieve aims and encourage communication about sexual health that helped communities develop knowledge and work towards better sexual health and wellbeing. Reviewing the strengths, challenges and/or lessons learned from each activity is useful to help adapt models for implementation in other PICTs [41]. Two themes identified from the analysis show what can help programs work: *building relationships* and *managing complex contexts*.

### Building relationships

Building relationships was reported as important for programs to meet objectives across all of the reviewed literature. Eight of the nine publications stated the benefits of establishing partnerships with a range of government and non-government organisations and agencies [32–38,40]. Programs without acceptance or consensus among power-holders in the community have a greater risk of failure [36]. Robust, collaborative relationships facilitated engagement and whole of community understanding of program aims, promoted advocacy and in some instances, generated support in materials, ongoing training and funding [34–37,40]. Relationships with agricultural and extractive industry partners in rural communities provided influential community members, including pastors’ wives and women leaders, with vital information about sexual health to be disseminated and reinforced in their communities on a continuing basis [35]. Partnerships with health service providers and centralised organisations were also reported as informing the preparation and implementation of national sexual health policy to affect change [38].

Four publications reported the importance of building and maintaining relationships with village community groups to engage and promote involvement in program activities [33,37–39]. This was particularly appropriate between educators, artists and local leaders to negotiate for respectful adaptation of sacred/private performance for the public space [39]. Partnerships that engage and inform were also useful for increasing local involvement in projects taking a participatory action research approach, and for trainee retention [38]. Across PICTs, relationships underpin social life and ongoing relationships with outsiders are valued by the community [38]. The most successful programs nurture and sustain community connections over time [34,35,38].

### Managing complex contexts

Analysis of the literature showed approaches that accounted for the diverse knowledge systems and identities within social structures and systems. A key factor was consideration of how the wider social- and context-shaped understandings and actions and connected participant experiences with their social and geographical location [32]. In seeking to increase awareness of sexual health issues and achieve behaviour change, authors acknowledged the tension between Western health promotion models that focus on the individual and internal change processes, and the collective orientation of Pacific societies, where family and community relationships are often the driver for change [32,36–39]. Success is more likely when individuals and communities themselves recognise the need, and make the choice, for change [36].

Several programs reported using approaches informed by existing models. Three studies adapted methodologies informed by Freire’s critical pedagogy and Boalian performance interventions to help participants explore the social, emotional and economic factors of an issue at community and individual levels [32,37,38]. The *Suiga/Change* program adapted Theatre of the Oppressed (TO) methodology to the Samoan setting, with participants using the TO technique of image theatre to embody personal, critical reflection of conflict and behaviours within intimate relationships [32]. The *Life Drama* program was situated within Theatre for Development (TfD), a participatory intervention for meaningful behaviour change used in HIV/AIDS prevention; improvised for the PNG setting with elements of Indigenous performance, *Life Drama* is underpinned by social, structural and environmental theories [38]. In recognition of the limitations of TfD for cross-cultural exchange, folk opera was identified by the *Life Drama* team as a way to connect TfD techniques with culturally-rich forms of PNG folk performance, enhancing the narrative about sexual health with performance traditions specific to time and place [39]. Also founded upon Freire’s critical pedagogy, the *Touching the Untouchables* (TTU) VHV training program was supplemented with community health evangelism training tools adapted to reflect the strong Christian beliefs of the local PNG setting [37]. Integration in content and methodology enabled empowerment and self-reliance at individual, collective and social levels, leading to reported change in gender relations, increased levels of leadership, and more effective ways of collective problem-solving and decision-making regarding health [37].

Community development principles informed the *Stret Toker* program [34]. Considerations of gender, peer education and community engagement contributed to the program’s theoretical framework, articulated in the sexual health promotion role of CEWs [34]. Training aimed to build understanding of the multifaceted social and cultural practices that increase an individual’s vulnerability to STIs, while adopting a rights-based approach to condom promotion: this could lead to sometimes difficult discussions among staff with differing cultural beliefs and biases about sexual health topics [34]. Sexual and reproductive health rights were core to the *Awareness Analysis Action* health worker practice manual [36]. Information within the manual, gathered from Pacific and international organisation policies and documents, provides an ethical frame of conduct for sexual health training programs in PICTs. Activity cards and practical strategies for advancement of SRHR offer multiple lenses through which programs can develop robust content and delivery in regard to marginalised communities. The emerging and promising practices reviewed described what worked and for whom. They illustrate the potential of training approaches that reflect the complexity of lived contexts in PICTs; they also provide *meri lidas*, valued for their knowledge and wisdom [2], with opportunities to engage with community members in informed discussions about sexual health issues.

## Discussion

This scoping review is the first to identify and evaluate community-based sexual health training in PICTs. This is important as the nature of evidence, although limited, provides an opportunity to inform and design sexual health training programs appropriate for settings within local communities in PICTs. Specifically, the findings provide a framework for developing training for an identified group of *meri lidas*, to deliver information appropriately and enable them to capably provide sexual health and wellbeing advice upon return to their local communities [42].

Context informs the link between health knowledge and health behaviour; understanding contextual elements is crucial to the development of effective interventions for behaviour change [43]. Findings suggest that social and cultural context drives program content and the means of program delivery in PICTs. The majority of reviewed studies focused on interventions for HIV and STIs. Papua New Guinea has the highest rate of HIV in the Pacific region [44], while in Samoa, rates of STIs including chlamydia and gonorrhea, remain endemically high [45]. These health challenges reflect the importance of addressing sexual health issues for particular community groups in PICTs and aligns with the specific information needs of the *meri lidas*.

Programs also sought to respond to issues of gender inequality, vital to improve SRHR for all, regardless of gender identity and sexuality [46] and necessary to achieve the Sustainable Development Goals of healthy lives and gender equality [47]. A gender-transformative approach in health is one that ‘addresses the causes of gender-based health inequities by including ways to transform harmful gender norms, roles and relations … to promote gender equality and foster progressive changes in power relationships between women and men’ [48,p.136]. However, this level of gender responsiveness may be difficult to achieve as it requires open and honest renegotiation of gendered norms, responsibilities and relations [48]. Evidence from a recent systematic review showed few gender-transformative interventions for SRHR engaged men/boys [46]. The inclusion of men/boys in sexual health programs of itself does not promote gender equality: interventions require explicit attention to gender inequalities to uphold women’s rights and autonomy [46]. A balance between engaging men and challenging them to question gender inequality is needed to achieve real gender-transformation [46]. Recently published principles and best practices for engaging males in programs preventing violence against women in the Pacific are grounded in the lived experiences of women/girls; these tenets recognise that men/boys can play a key role as allies and advocates to transform gender relations on an individual and community basis and promote policy change [49]. In PICTs where concepts of gender can present as a barrier to program delivery, consideration of how gender is distinguished in specific settings can aid in developing programs that have the ability to engage people from diverse backgrounds within the community to influence change.

The benefits of partnering with faith-based organisations to engage participants and garner support to achieve sexual health outcomes were reported in the reviewed literature. In countries where religion shapes individual and collective identity, the interconnectedness of spirituality and health decision-making is significant and should be considered in health promotion with specific population groups [50]. With unique cultural resources, community connection and the ability to reach a diverse range of people, churches can play an important role in health promotion and in the recruitment and training of lay people for behaviour change [51]. For example, the *Trusted Messenger* approach for HIV prevention in Zambia provided religious leaders with biomedical information, to better understand HIV and effectively address the health concerns of members of their communities [52]. Programs such as *Trusted Messenger* provide evidence of translating new knowledge into practice, and may be adapted for training *meri lidas* returning to communities where members hold a strong faith.

Several programs in this review incorporated elements of storytelling – theatre, poetry, metaphor, song and dance, reflecting the rich oral traditions of PICTs. Stories are effective educational tools as they speak of the human experience as a source of information and wisdom in the telling and retelling [53,54]. Performative interventions in sexual and reproductive health have been used in other resource-limited communities with primarily oral cultures and low literacy levels: for example in Uganda, for HIV [55], maternal and child health [56], and HIV awareness [57]; in South Africa, for sexual health communication [58] and HIV stigma [59]; and in China, for HIV awareness and safe sex promotion [60]. Once viewed by scholars as static practices, traditional performative practices are now understood as contextual, strategic, dynamic and inherently complex [53,61]. Following the teachings of Freire and Boal, the use of theatre to educate, liberate and give community a voice to discuss specific concerns and health challenges can highlight information gaps and provide insight into how health information can be customised to address community needs [57]. In PICTs, where quality health information is often unavailable, communities rely on stories to make decisions regarding health behaviour [62]. According to Silver [56], storytelling as a method to effect health behaviour change requires only imagination and understanding of the cognitive frame of a particular community. Silver states:

True health communication occurs only by transforming health knowledge into messages that can be readily understood, accepted, and acted upon by the intended audience. The time-honored oral traditions of songs and storytelling offer inexpensive, culturally appropriate ways of bringing health messages to life by infusing them with the active participation and lively spirit of the people for whom they are intended. As such, songs and storytelling can play a fundamental role in the process of continuing education, which is the key to the long-term sustainability of health promotion efforts [56,p.58].

While cultural considerations may prevent open and direct discussion of sexual health and wellbeing, programs using storytelling methods offer culturally safe spaces to discuss topics and concerns, share information and address issues. These spaces exist in localised contexts and require further exploration. Identifying and understanding these spaces can greatly enhance training programs that seek to educate and inform on matters of sexual health and wellbeing in PICTs.

The review identified emerging and promising practice in sexual health training in the Pacific, yet notably absent was robust evaluation of programs. Comprehensive evaluation of effectiveness would help researchers build on existing program design to achieve intended outcomes in specific settings [63,64]. Knowing the context in which a program is delivered and received is key to explicating how and why the program does or does not work for the targeted population, how it might be improved and how outcomes may differ when the program is implemented elsewhere or with a different group [65].

## Conclusion

Culture, religion and gender and their effects are important considerations when developing science-based training programs for sexual health and wellbeing in identified PICT communities. Modes of delivery that engage participants in socially responsive ways promote positive change and maximize program success. The review provides evidence to develop training for women to ensure relevance and appropriateness in diverse Pacific settings and to plan and implement robust evaluation. Women leaders with deep and ongoing connection to community would be well-placed to provide information and advice following sexual health training, translating new knowledge into practice for improved health and wellbeing in PICTs.

## Paper context

Women leaders in Pacific Island nations need access to sexual health information to provide appropriate advice about sexual and reproductive health issues to members of their communities. The characteristics of community-based sexual health training programs in Pacific Island nations are identified and evaluated, providing an evidence-base to inform the development of sexual health training relevant to Pacific women. Design and delivery of sexual health programs in PICTs should be tailored to local socio-cultural contexts.

## Appendix A. MEDLINE search strategy

**Table:**

1	Women/	14,789
2	Female/	8,841,613
3	Men/	3301
4	Male/	8,700,845
5	1 or 2 or 3 or 4	11,634,452
6	Sex Education	8851
7	train*.mp	592,918
8	educat*.mp	1,055,101
9	workshop*.mp.	40,764
10	instruct*.mp.	110,086
11	6 or 7 or 8 or 9 or 10	1,538,844
12	Exp Sexual Behavior/	108,484
13	sexual behaviour.mp.	5626
14	sexual behavio?r.mp.	87,512
15	Sexual Health/	1168
16	sexual health.mp.	10,972
17	Sexually Transmitted Diseases/	24,879
18	sexually transmitted infection*.mp.	14,643
19	12 or 13 or 14 or 15 or 16 or 17 or 18	162,310
20	exp Polynesia/	10,558
21	polynesia.mp.	2642
22	exp Melanesia/	6525
23	melanesia.mp.	1463
24	exp Micronesia/	2004
25	micronesia.mp.	1666
26	Pacific Islands/	3990
27	pacific island countries.mp.	312
28	(pacific island countries and territories).mp.	99
29	american samoa.mp.	406
30	Cook Islands.mp.	220
31	Federated States of Micronesia.mp.	265
32	fiji.mp.	2038
33	french polynesia.mp.	1025
34	guam.mp.	1366
35	Kiribati.mp.	209
36	Marshall Islands.mp.	308
37	nauru.mp.	159
38	New Caledonia.mp.	1649
39	niue.mp.	74
40	Northern Mariana Islands.mp.	137
41	Palau.mp.	435
42	Papua New Guinea.mp.	5445
43	Pitcairn Islands.mp.	8
44	pitcairn island.mp.	21
45	samoa.mp.	1192
46	solomon islands.mp.	842
47	tahiti.mp.	282
48	tokelau.mp.	96
49	tonga.mp	516
50	tuvalu.mp.	75
51	vanuatu.mp.	735
52	(Wallis and Futuna).mp.	44
53	20 or 21 or 22 or 23 or 24 or 25 or 26 or 27 or 28 or 29 or 30 or 31 or 32 or 33 or 34 or 35 or 36 or 37 or 38 or 39 or 40 or 41 or 42 or 43 or 44 or 45 or 46 or 47 or 48 or 49 or 50 or 51 or 52	27,748
54	5 and 11 and 19 and 53	74
55	Published after 2002	42
